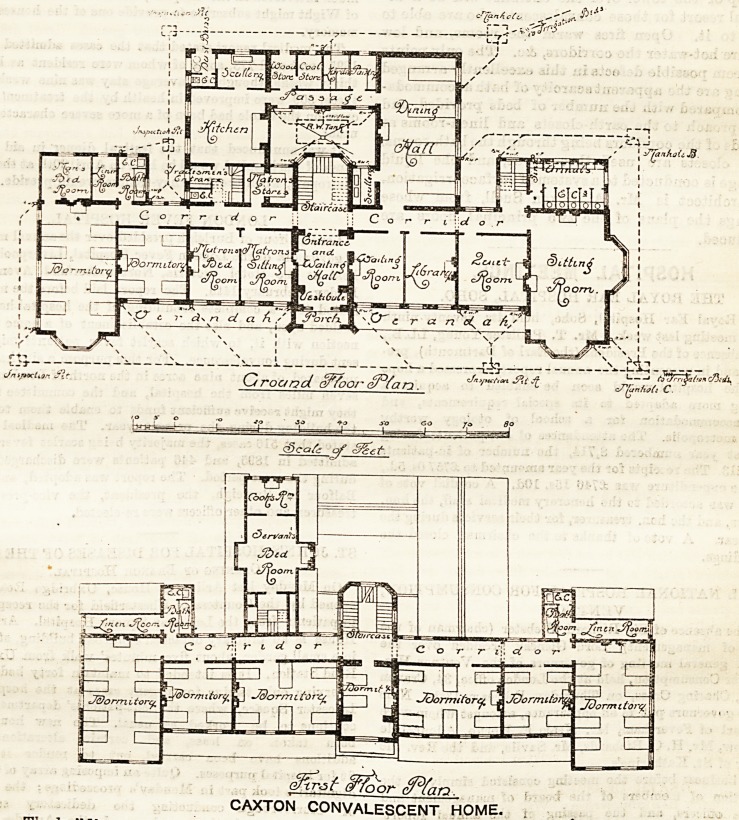# Construction Notes

**Published:** 1896-02-22

**Authors:** 


### THE INSTITUTIONAL WORKSHOP.

### CONSTRUCTION NOTES.

### THE CAXTON CONVALESCENT HOME, LIMPSFIELD, SURREY.

This institution is being erected at the expense of Mr. Passmore Edwards, for the benefit of the printing and allied trades, on high ground adjoining Limpsfield Common and overlooking the South Downs. The building consists of a two-storeyed block containing day-rooms and dormitories for fifty men. The site falls rapidly from north to south, and has given the architect an opportunity of obtaining at the south end of the main block, under the ground floor, a smoking or billiard-room, with cloak-rooms, lavatories, &c., and he has placed the general entrance for patients on this floor. The principal entrance is on the ground floor in the centre of the building, with a waiting-room well arranged on the right, so that visitors need not enter the patients' portion of the building. A wide well-lighted corridor leads on the right hand to a library, a "quiet room" (excellently placed), and a large sitting-room. A lobby at the end leads to a range of earth-closets and to the stairs down to the basement already referred to. The dining-hall (a one-storeyed building) runs back at right angles to the corridor, and is connected by a passage, on one side of a courtyard, with the kitchen and offices. To the left of the principal entrance are the matron's sitting and bed-rooms, with her store-room on the opposite side of the corridor, close to the trades' entrance and servants' stairs. Two dormitories, one for four and one for eight beds, with bath-room and earth-closet in proximity, and a small bed-room opening out of the larger dormitory—are arranged at the extreme end of the corridor. The main staircase, opposite the principal entrance, leads to a similar wide corridor on

CAXTON CONVALESCENT HOME.

the first floor, off which opens a succession of dormitories of various sizes—two for eight, two for four, two for six, and one for two beds, giving a minimum of 750 cubic feet to each bed. The servants' wing over the kitchen is entirely (and properly) cut off and approached by a separate staircase. A bath-room, earth-closet and linen-room are provided at each end of the corridor. Arrangements are made in the construction for additional rooms in the roof to accommodate twenty-two beds, and a "sun-room," lighted on all sides, at the top of the tower over the entrance will be a delightful resort for those convalescents who are able to climb to it. Open fires warm the rooms, and low pressure hot-water the corridors, &c. The only points that seem possible defects in this excellently arranged building are the apparent scarcity of bath accommodation compared with the number of beds provided, and the approach to the earth-closets and linen-rooms at the ends of the corridors being through the bath-rooms. Earth closets are used throughout, and the liquid rainage is conducted to a system of surface irrigation. The architect is Mr. A. Saxon Snell, from whose drawings the plans of the two principal floors are reproduced.

## Figures and Tables

**Figure f1:**